# A further insight into the biosorption mechanism of Au(III) by infrared spectrometry

**DOI:** 10.1186/1472-6750-11-98

**Published:** 2011-10-27

**Authors:** Zhongyu Lin, Yiwen Ye, Qiaoling Li, Zhenling Xu, Miao Wang

**Affiliations:** 1 State Key Laboratory of Physical Chemistry of Solid Surfaces and Department of Chemistry, College of Chemistry and Chemical Engineering, Xiamen University, Xiamen 361005, PR China; 2 Department of Chemical and Biochemical Engineering, College of Chemistry and Chemical Engineering, Xiamen University, Xiamen 361005, PR China

## Abstract

**Background:**

The interactions of microbes with metal ions form an important basis for our study of biotechnological applications. Despite the recent progress in studying some properties of Au(III) adsorption and reduction by *Bacillus megatherium *D01 biomass, there is still a need for additional data on the molecular mechanisms of biosorbents responsible for their interactions with Au(III) to have a further insight and to make a better exposition.

**Results:**

The biosorption mechanism of Au(III) onto the resting cell of *Bacillus megatherium *D01 biomass on a molecular level has been further studied here. The infrared (IR) spectroscopy on D01 biomass and that binding Au(III) demonstrates that the molecular recognition of and binding to Au(III) appear to occur mostly with oxygenous- and nitrogenous-active groups of polysaccharides and proteins in cell wall biopolymers, such as hydroxyl of saccharides, carboxylate anion of amino-acid residues (side-chains of polypeptide backbone), peptide bond (amide I and amide II bands), etc.; and that the active groups must serve as nucleation sites for Au(0) nuclei growth. A further investigation on the interactions of each of the soluble hydrolysates of D01, *Bacillus licheniformis *R08, *Lactobacillus *sp. strain A09 and waste *Saccharomyces cerevisiae *biomasses with Au(III) by IR spectrometry clearly reveals an essential biomacromolecule-characteristic that seems the binding of Au(III) to the oxygen of the peptide bond has caused a significant, molecular conformation-rearrangement in polypeptide backbones from β-pleated sheet to α-helices and/or β-turns of protein secondary structure; and that this changing appears to be accompanied by the occurrence, in the peptide bond, of much unbound -C=O and H-N- groups, being freed from the inter-molecular hydrogen-bonding of the β-pleated sheet and carried on the helical forms, as well as by the alternation in side chain steric positions of protein primary structure. This might be reasonably expected to result in higher-affinity interactions of peptide bond and side chains with Au(III).

**Conclusions:**

The evidence suggests that the polypeptides appear to be activated by the intervention of Au(III) via the molecular reconformation and in turn react upon Au(III) actively and exert profound impacts on the course of Au(0) nucleation and crystal growth.

## Background

The elucidation of the mechanisms active in metal biosorption is essential for the successful exploitation of microbial resources in the fields of bioremediation [1,2] and biosynthesis [3-5]; and the roles the microbes playing in the biosyntheses have attracted close attention. Gold nanoscale material is one of the significant precious metal catalysts; and the microbe-Au(III) interactions should be one of the key factors in producing effects on Au(0) nanoparticle growth and regulation. A report series on the characterizations of adsorption and reduction of noble metals by the biomasses of *Bacillus megatherium *D01 and waste *Saccharomyces *(*S*.) *cerevisiae *have once been made [6-12]; still, the additional data are needed to address the impacts of polysaccharides and protein primary structure (sequences of amino-acids joined to a covalent polypeptide backbone and/or side-chain steric positions) and secondary structure (conformations of periodic arrays of inter- and/or intra-molecular hydrogen-bonding between -N-H and O=C- groups of polypeptide backbone units) of microbial biomasses on their interactions with gold. The soluble D01 hydrolysate (filtrate) is an important composition of the biomass and plays a key role in the interaction with Au(III). The IR absorptions of the filtrate binding Au(III) are closely correlated to that of the D01 biomass binding. In that the former can get rid of any insoluble materials that may interfere with the IR absorptions studied to exhibit the subtle differences between the bands of the filtrate and that binding and mirrors the conformational changes in protein primary and secondary structures directly, thus revealing the molecular mechanisms of protein binding Au(III) more sensitively than the latter; this soluble hydrolysate complements the D01 biomass perfectly. Herein, we present, based on the previous studies, a further investigation into the microcosmic process of Au(III) biosorption by the resting cell of *Bacillus megatherium *D01 biomass and its soluble hydrolysate using IR and other spectroscopic methods.

The strain D01 was screened out from different bacterial strains that were isolated from the soils of mining areas. It has a strong ability to adsorb and reduce Au(III) as well as a good resistance to the action of Au(III). It is easy to gain and culture and still grows well in a medium containing 600 mg/l Au(III) [7]. The adsorptive capacity of the resting cell of this strain for Au(III) approached 300 mg/g when the biomass was suspended in a 0.5 mM auric chloride acid (AuCl_3_·HCl·4H_2_O) aqueous solution (250 mg/l) at pH 3.0 and 37°C for 2 hours (h). The strain D01 is part of the Gram-positive bacteria and identified as *Bacillus megatherium*.

The earlier pH dependent experiments showed that the adsorptive capacity of the D01 biomass for Au(III) seems to have a direct bearing on pH with the maximum adsorption near pH 3.0 [10,12]. When pHs < 3.0, most of the proton may compete with Au(III) for binding sites of active groups on cell walls, so that the adsorptive capacity of the biomass for Au(III) decreases with the pH falling; when pHs > 3.0, it causes the precipitation of gold hydroxides, which may disturb the adsorption and also incurs a reduction in the adsorptive capacity. This trend in pH dependence suggests an ion exchange mechanism of the adsorption of Au(III) onto the biomass [13]. The initial adsorption process dominates the events of Au(III) binding, reduction and nucleation. And the information on pH profile for Au(III) adsorbed by the biomass may provide a useful basis for discussion as to the effect of pH on the regulation of Au(0) nanoparticle sizes by functional groups of the biomass.

The state of Au(III) bioreduced to elemental Au(0) was investigated using X-ray powder diffractometry (XRD). The analyses for sulphur and glucose contents in the D01 biomass were performed by energy-dispersive X-ray analysis (EDX) and ultraviolet-visible (UV-vis) spectrophotometry respectively. The molecular mechanisms responsible for the effects of saccharides and proteins both from the D01 biomass and from the soluble hydrolysates of D01 and some other microbial (R08, A09 and waste *S. cerevisiae*) biomasses on their interactions with gold were further studied by means of IR spectrometry.

## Methods

### Biosorbent preparation

The biosorbent was prepared in accordance with a reported method [7]. The strain D01 was cultivated in an aqueous solution containing beef gels, peptone, salt, etc. and harvested at its growth stage. The biomass was obtained by centrifugation at 3500 rings per minute (rpm) for 15 min on a centrifuge to remove the upper culture medium, dialyzed against deionized water to remove any soluble substances (including metal ions) that could interfere with the Au(III) studied, and dried under vacuum at 37°C. The dried biomass was ground into powder and it was then stored in a desiccator for use.

### XRD examination

A sample of the above D01 biomass powder suspended in the auric chloride acid aqueous solution at pH 3.0 and 37°C was shaken at 130 rpm for 24 h, followed by being dried at ambient temperature. The dried sample was ground into powder and it was then examined using a Rigaku D/max-rC X-ray diffractometer (Japan). The pattern was recorded by Cu Kα radiation with λ of 1.5418 Å and graphite monochromator filtering wave, and by scanning at tube voltage of 40 kV and tube current of 30 mA in the region of 30°- 90° at 6°/min with incident-beam 2θ.

### EDX and UV-vis experiments

EDX analysis: Triplicate samples of the powdered D01 biomass in small quantity were analyzed for sulphur content using an ISIS 300 INCA energy dispersive X-ray analysis system (England).

UV-vis spectrophotometry: Two 30 mg samples of the powdered D01 biomass were washed with diluted HCl and then suspended, respectively, in 6 ml of diluted HCl with a final biomass concentration of 5 mg/ml and the pH adjustment at 3.0. Two milliliters of the biomass suspension (10 mg biomass) was placed into six test tubes, followed by centrifugation to remove the supernatants. A 2 ml aliquot of deionized water at pH 3.0 was added to each of the six samples of the biomass; and they each were then stirred up, followed by shake at 130 rpm in an incubator at 37°C. Two sections of three samples each for the respective reaction times of 10 min and 24 h were centrifugalized at 3500 rpm for 6 min. The supernatants of the tubes (soluble hydrolysate of the biomass) were each analyzed for glucose content by a phenol-sulfuric acid method [14,15], reading the absorbances at 488 nm in a 752 UV-visible spectrophotometer (China).

### IR spectrometry

For IR comparative study, two sections of two samples each: (i) D01 biomass (ii) that challenged with Au(III) at pH 3.0 and 37°C for 24 h (iii) soluble hydrolysate (filtrate) of D01 biomass on hydrolysis for 24 h and (iv) that reacted with Au(III) at pH 3.0 and 37°C for 2 h, and an additional three sections of two samples each: (v) filtrate of *Bacillus licheniformis *R08 biomass on hydrolysis for 24 h (vi) that reacted with Au(III) at pH 3.0 and 37°C for 2 h (vii) filtrate of *Lactobacillus *sp. strain A09 biomass on hydrolysis for 24 h (viii) that reacted with Au(III) at pH 3.0 and 37°C for 2 h (ix) filtrate of waste *S. cerevisiae *biomass on hydrolysis for 24 h and (x) that reacted with Au(III) at pH 3.0 and 37°C for 2 h, were analysed. The samples for IR examination were dried under vacuum at 37°C to thoroughly eliminate the liquid water that can strongly interfere with the IR absorptions and then prepared by pressing each dried KBr-sample mixture, which contains about 50 mg finely ground KBr powder mixed intimately with 0.25 - 0.5 mg powdered sample, into a transparent disc with 0.2 mm in thickness under a fixed pressure of about 3000 kg/cm^2^. Then the pellets were each determined on a Nicolet-740SX FT-IR spectrophotometer (USA) with a MCT-B detector. The spectra were recorded in the range 4000 - 625 cm^-1 ^at a resolution of 4 cm^-1 ^with 32 scans. The IR experiments were each performed in triplicate for the purposes of quality control and statistics.

## Results and Discussion

### Adsorption of Au(III) onto D01 biomass

Generally, the surface of the D01 biomass suspended in aqueous solution at pH 3.0 is positively charged due to the protonated oxygenous- and nitrogenous-active-groups of cell wall biopolymers, e.g.:

$$\begin{array}{c} \text{the free hydroxyl},\;\text{-C}\text{-OH+}{\text{H}}^{+}\to \text{-C-}{\text{OH}}_{2}^{+},\;\text{etc}\text{.;}\\ \text{the carboxylate anion},\\ \;\;\;\text{-C}{\left(\underline{---}\text{O}\right)}_{2}^{-}+2{\text{H}}^{+}\to \text{-C}{\left(\underline{---}\text{O}\right)}_{2}{\text{H}}_{2}^{+},\;\text{etc}\text{.;}\\ \text{the peptide bond},\;\text{-HN-C=O}+\;{\text{H}}^{+}\to \text{-HN-C=O}{\text{H}}^{\text{+}}\\ \text{and}∕\text{or }\text{-HN-C=O}+\text{2}{\text{H}}^{+}\to {\text{-NH}}_{2}^{+}{\text{-C=OH}}^{+},\text{etc}. \end{array}$$

And the auric chloride acid in aqueous solution generally forms ${\text{AuCl}}_{4}^{-}$ anion, so it was rapidly adsorbed on the surface of the biomass by these protonated-active-groups via electrostatic interactions when meeting with the microbe. Greene et al. [16] noted that the release of the chloride ion from the adsorbed ${\text{AuCl}}_{4}^{-}$ occurred simultaneously with the adsorption of gold within the time frame of the experiments by in situ determinations of the free chloride ion, and pointed out that the mechanism of this reaction is thought to involve the initial formation of ion pairs between negatively charged ${\text{AuCl}}_{4}^{-}$ and positively charged protonated-active-groups, followed by the elimination of an ion pair of chloride and proton. The adsorption species of Au(III) by some active groups on the biomass are briefly represented as follows:

$$\begin{array}{c} {\text{AuCl}}_{4}^{-}+4\left({\text{-C-OH}}_{2}^{+}\right)\to \text{Au}\left(\text{III}\right)\cdot {\left(\text{-C-OH}\cdot \text{HCl}\right)}_{4}\\ \;\;\;\;\;\;\;\;\;\to \text{Au}\left(\text{III}\right)\cdot {\left(\text{-C-OH}\right)}_{4}+\text{4HCl}\\ {\text{AuCl}}_{4}^{-}+2\left[\text{-C}{\left(\underline{---}\text{O}\right)}_{\text{2}}{\text{H}}_{2}^{+}\right]\to \text{Au}\left(\text{III}\right)\cdot {\left[\text{-C}{\left(\underline{---}\text{O}\right)}_{2}^{-}\cdot \text{2HCl}\right]}_{2}\\ \;\;\;\;\;\;\;\;\;\to \text{Au}\left(\text{III}\right)\cdot {\left[\text{-C}{\left(\underline{---}\text{O}\right)}_{2}^{-}\right]}_{2}+4\text{HCl}\\ {\text{AuCl}}_{4}^{-}+4\left(\text{-HN-C=O}{\text{H}}^{+}\right)\to \text{Au}\left(\text{III}\right)\cdot {\left(\text{-HN-C=O}\text{HCl}\right)}_{4}\\ \;\;\;\;\;\;\;\;\;\to \text{Au}\left(\text{III}\right)\cdot {\left(\text{-HN-C=O}\right)}_{4}+4\text{HCl}\\ {\text{AuCl}}_{4}^{-}+2\left({\text{-NH}}_{2}^{+}\text{-C=O}{\text{H}}^{\text{+}}\right)\to \text{Au}\left(\text{III}\right)\cdot {\left(\text{-HN-C=O}\cdot 2\text{HCl}\right)}_{2}\\ \;\;\;\;\;\;\;\;\;\to \text{Au}\left(\text{III}\right)\cdot {\left(\text{-HN-C=O}\right)}_{2}+4\text{HCl,}\;\text{etc}\text{.} \end{array}$$

The mechanism of ion exchange apparently prevails in this adsorption [13]. The resulting adsorbate on the cell walls should be mainly the Au(III); and it plays a leading role in the interactions with the biomass. The free Au(III) species should still take the form of ${\text{AuCl}}_{4}^{-}$ anion remaining in aqueous solution; and the ${\text{AuCl}}_{4}^{-}$ anion therefore is neglected while discussing the Au(III) binding case.

### XRD characterization of Au(III) biosorption

The previous transmission-electron-micrograph of Au(III) challenging D01 biomass at ambient temperature for 2 h exhibited the distribution of Au(0) nanoparticles with the sizes of a few to more than one hundred nanometers (nm) on the cell surfaces [12,17]; and the photoelectron spectrum of that for 48 h gave a spectrum with the only peaks of Au(0) (4f, 5/2) and Au(0) (4f, 7/2) [7]. Further analysis of the biomass reacting on Au(III) for 24 h by XRD has displayed a pattern with peaks corresponding exactly to those of elemental Au(0). The results lead to the suggestions that the biomass has reduced the Au(III) to Au(0); and the biomass itself must have served as a catalyst enzymatically catalyzing both the bioreduction of Au(III) to Au(0) and the formation of Au(0) nanoparticles, as well as playing a role in regulating and stabilizing the nanoparticles to a certain extent besides as an electron donor during this biosorption.

### Analyses for sulfur and glucose contents in D01 biomass

The average sulfur content is about 0.38% of the D01 biomass and balances only about 0.17% of the atomic content by EDX (analysis). While thiol ligands (-SH and -SCH_3_), such as cysteine [18] and methionine [19], are known to reduce Au(III) to Au(I) or Au(0), the content of the sulfur atom is no more than 0.17% in the biomass. This shows that both cysteine and methionine of amino-acid residues (side-chains) in the biomass are very small in quantity.

Whereas the average glucose content of the soluble hydrolysate of polysaccharides, analyzed by UV-vis spectrophotometry, corresponds to 2.34% of the D01 biomass on hydrolysis for 10 min and to 3.35% of that for 24 h. And the filtrate of the biomass generally contains other reducing sugars, such as oligosaccharides, dioses, monoses, etc. besides the glucose; thus, the quantity of all the reducing sugars in the soluble hydrolysate must be much larger than 2.34% and 3.35% on hydrolysis for 10 min and 24 h respectively. The result indicates that the content of all the reducing sugars are far more than that of both cysteine and methionine in the biomass; and implies that the reducing sugars may serve as the main electron donor in situ reducing Au(III) to Au(0) in this system. The glucose content on hydrolysis for only 10 min has nearly reached 70% of that for 24 h, showing that the hydrolysis of the polysaccharides of the biomass is a rapid process and may mostly limit on the cell wall surfaces. This might facilitate the following bioreduction of the bound Au(III) to Au(0) and then the formation of gold nanoparticles.

### IR characterization of Au(III) biosorption

#### Binding of Au(III) to D01 biomass

The biomacromolecular recognition of and binding to Au(III) appear to be primarily associated with oxygenic- and nitrogenous-active groups of polysaccharides and proteins (primary and secondary structures) of microbial biomasses. For better understanding of the molecular mechanisms of saccharides and proteins both reacted with Au(III), the IR comparative study of the D01 biomass and that challenged with Au(III) has been performed.

There is experimental evidence that the interaction of AuCl_4_^- ^with the algal biomass involves the rapid reduction of Au(III) to Au(I), followed by a slow reduction to Au(0) [16]. Based on this premise and the preceding mention of the main Au(III) adsorption speciations on the biomass at the beginning of the biosorption as well as on valence bond theory and IR spectrographic study, certain binding speciations of Au(III), Au(I) and Au(0) with the functional groups of the cell wall biopolymers are speculated in rough. The electronic configuration of the valence shell of Au(0) is 5*d*^10^6*s*^1^; for Au(I) it is 5*d*^10^6*s*^0^, having *sp*-hybridized orbitals; and for the Au(III) it is 5*d*^8^6*s*^0^, bearing *dsp^2^*-hybridized orbitals. From this viewpoint it appears that Au(III) tends to take a four-coordinate, planar complexation with donor atoms of active groups, such as oxygen and/or nitrogen; in this case, each of the four coordinating atoms (O and/or N) provides one of their "unshared" electron pairs, i.e. lone pair electrons, to the four unfilled 5*d*, 6*s *and two 6*p *orbitals of Au(III) to bring the four-covalent-coordinate, planar complexation with Au(III). For Au(I) it is 5*d*^10 ^ion and tends to assume its 6s and 6p orbital hybridization with two donor atoms of O and/or N to form a two-coordinate, linear complexation. And for Au(0), based on its 5*d*^10 ^atom, it may well retain the two-coordinate complexation as the Au(I) binding.

**Figure 1:**
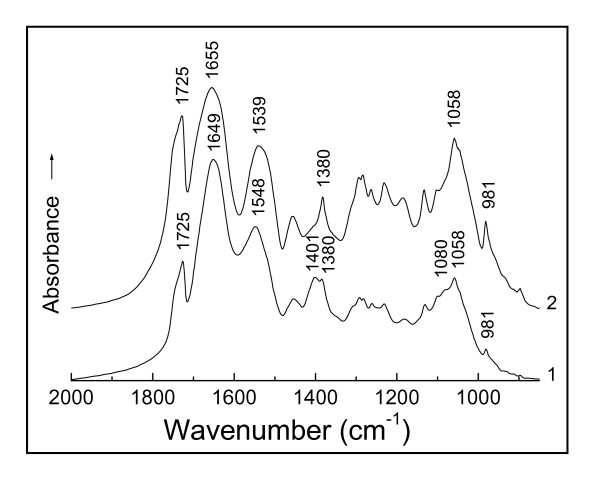
**IR spectra of (1) *Bacillus megatherium *D01 biomass and (2) that challenged with Au(III) at pH 3 for 24 h**.

The IR spectra of the D01 biomass and that reacted with Au(III) are shown in Figure 1*, curve 1 and 2 [10] respectively. The curve 1 shows a shoulder peak at 1 080 cm^-1 ^corresponding to a coupled vibration involving C-O stretching and O-H deformation modes (υ_C-O _+ δ_O-H_) of the free hydroxyl (-C-O-H) of saccharides [20]; and the band vanished after the biomass in contact with Au(III) (curve 2). This is most likely due to binding or chelation of Au(III) to the oxygen of the hydroxyl, implying the Au(III) binding speciation of the four-coordinate, planar complexation as (-C-HO)_2_:Au(III):(OH-C-)_2_, etc. In this case, the increases in both the bond-lengths of C-O and O-H of the hydroxyl occur, thereby incurring a red-shift (low-frequency shift) of this shoulder peak [20] and resulting in the disappearance of this band. Then the Au(III) binding species were rapidly transformed into the Au(I) species of the two-coordinate, linear complexation as -C-HO·Au(I)·OH-C-, etc., followed by a slow transition to Au(0) species as -C-HO·Au(0)·OH-C-, etc. with the proceeding of Au(III) being reduced to Au(0). A similar change for the same reason is observed at 1 401 cm^-1 ^(curve 1) arising from the symmetrical $\text{-C(}\underline{---}{\text{O)}}_{2}^{-}$ stretching band of the carboxylate anion (-COO^-^) of amino-acid residues of polypeptide backbones [20,21]. A red-shift of this absorption from 1 401 cm^-1 ^to 1380 cm^-1 ^(curve 2) is attributable to the chelation of Au(III) with the oxygen of the carboxylate anion, being speculated the Au(III) binding speciation by coordinating the carboxylate anion [22], such as  (the four coordinating oxygen from two ligands in the former and that from four in the latter), etc.; and then they rapidly changed to Au(I) binding species, such as  (the two donor oxygen from two ligands in the former and that from one in the latter), etc., followed by a slow change to Au(0) species, such as  , etc. The absorption at 1 548 cm^-1 ^results from a coupled vibration involving C-N stretching and N-H bending modes (υ_C-N _+ δ_N-H_) of the C-N-H group from the peptide bond (-HN-C=O), i.e. of the amide II band [20,21,23,24]; a red-shift of this band observed from 1 548 to 1 539 cm^-1 ^after the biomass in contact with Au(III) is due to the binding of Au(III) to the nitrogen of the amide II band [23]. Another blue-shift (high-frequency shift) from 1 649 to 1 655 cm^-1 ^is ascribable to the complexation of Au(III) with the oxygen of the carbonyl (C=O) from the peptide bond, i.e. of the amide I band [23]. Both the frequency shifts of amide I and II bands are assumed the occurrence of the coordination of Au(III) with four donor atoms of two oxygen and two nitrogen from two and/or four ligands (peptide bond), such as -HN-C=O:Au(III):O=C-NH- and/or [O=C-NH-]_2_:Au(III):[O=C-NH-]_2_, etc.; and then it was quickly turned into the two-coordinate complexation of Au(I) with both O and N atoms of two and/or one ligand, such as [O=C-NH-]·Au(I)·[O=C-NH-] and/or Au(I):O=C-NH-, etc., followed by slowly changing to Au(0) binding species, such as [O=C-NH-]·Au(0)·[O=C-NH-] and/or Au(0):O=C-NH-, etc. The absorption of the amide I band seems to be closely connected with protein secondary structure. This frequency blue-shift of the amide I band by Au(III) binding is thought to involve the reconformation of the polypeptide backbones, namely a conformational change in the protein secondary structure after the biomass in contact with Au(III); a detailed explanation of this will be given later.

On comparing both intensities of the carbonyl absorptions of the carboxyl at 1 725 cm^-1 ^between the D01 biomass (I_1725_^0^) and that binding Au(III) (I_1725_^b^) in Figure 1, bands at 1 649 cm^-1 ^(I_1649_) and 1 655 cm^-1 ^(I_1655_) due to their respective carbonyl absorptions of the peptide bond (amide I band) were each selected as reference peaks for the semiquantitative assessment of the carboxyl [6] at curves 1 and 2, respectively. Based on the mathematical viewpoint, the quantity of the carbonyl absorptions of the peptide bond is far larger than that of the carboxyl and can generally be regarded as an almost changelessness in its absorption intensity while binding; therefore the value of the I_1655 _is thought to be nearly equal to that of the I_1649 _under the above conditions of sample handling. And the rate of I_1725_^b^/I_1655 _is appreciably larger than that of I_1725_^0^/I_1649 _in this figure, the enhancement of the carboxyl absorption of the D01 biomass binding (curves 2) being thus confirmed. The following comparisons between the two absorption intensities of biomass and that binding in IR spectra have all been made in accordance with this method and taken seriously.

#### Binding of Au(III) to filtrates of D01 and some other biomasses

**Figure 2:**
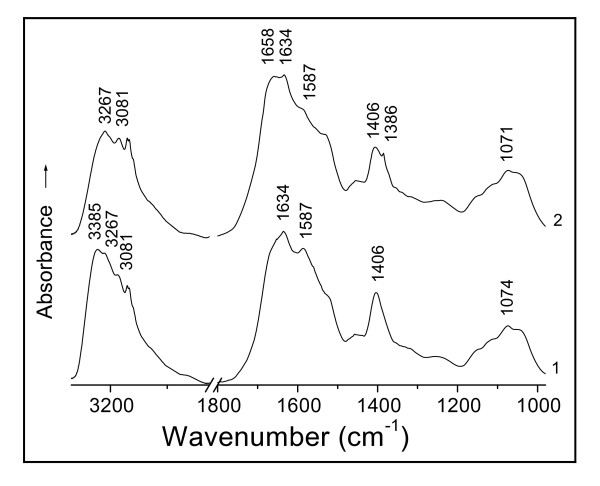
**IR spectra of (1) soluble hydrolysate of D01 biomass on hydrolysis for 24 h and (2) that challenged with Au(III) at pH 3 for 2 h**.

IR spectra are of additive property. The bands of the D01 biomass reacted with Au(III) shown in Figure 1, curve 2 involve the absorptions of both soluble and insoluble D01 hydrolysate binding Au(III). The soluble part is an important composition of the biomass and plays a key role in the interaction with Au(III). Yet the content of the soluble hydrolysate is much less than that of the insoluble, so the absorptions of the former have been obscured by those of the latter whose spectrum is closely similar to that of the D01 biomass. In order to remove any insoluble materials that may interfere with the IR absorptions studied, the filtrate and that binding Au(III) have been recorded by IR respectively (Figure 2, curve 1 and 2); they show closely correlated to the D01 biomass and that binding. Of the biomass and its filtrate, the latter has succeeded in avoiding the overlapping of the amide I band region by the insoluble materials to display the subtle differences between the absorptions of the carbonyl and that binding, and directly reflects the conformational changes in polypeptide backbones and in their side chain steric positions, thus revealing the molecular mechanisms of protein binding Au(III) more sensitively than the former; this filtrate complements the biomass perfectly. Therefore the soluble hydrolysates of D01 and some other microbial (R08, A09 and waste *S. cerevisiae*) biomasses and those binding have each been used to further illustrate the mechanisms of hydroxyl, carboxylate anion and peptide bond of the biomasses reacted with Au(III).

**Figure 3:**
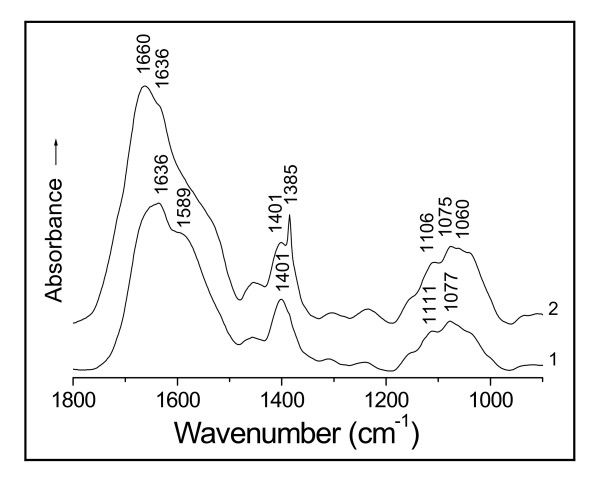
**IR spectra of (1) soluble hydrolysate of *Bacillus licheniformis *R08 biomass on hydrolysis for 24 h and (2) that challenged with Au(III) at pH 3 for 2 h**.

**Figure 4:**
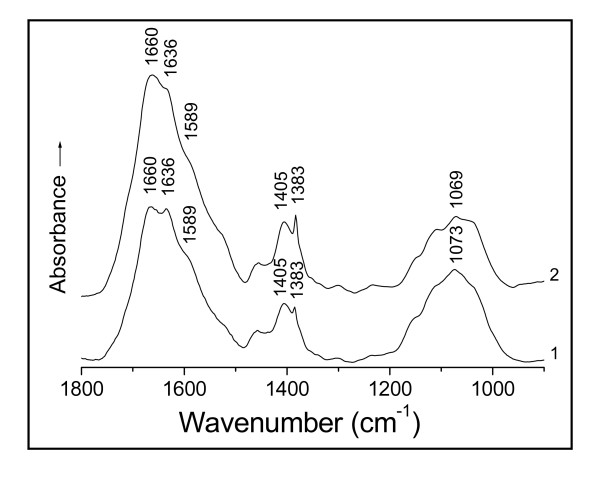
**IR spectra of (1) soluble hydrolysate of *Lactobacillus *sp. strain A09 biomass on hydrolysis for 24 h and (2) that challenged with Au(III) at pH 3 for 2 h**.

**Figure 5:**
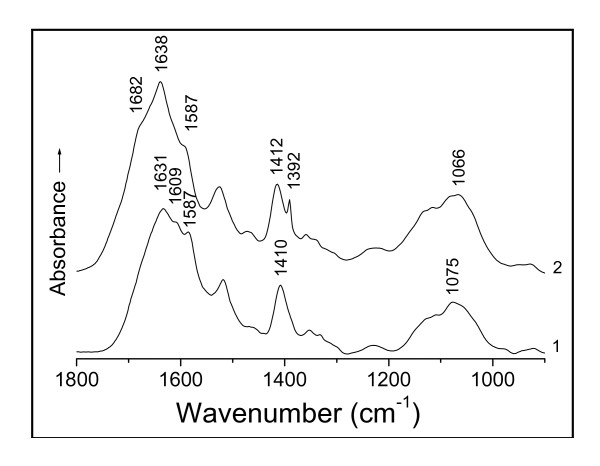
**IR spectra of (1) soluble hydrolysate of *Saccharomyces cerevisiae *waste biomass on hydrolysis for 24 h and (2) that challenged with Au(III) at pH 3 for 2 h**.

The amino-acids don't show any absorptions of the N-H stretching bands in the normal 3 500 - 3 300 cm^-1 ^range; and the polypeptides only appear medium and weak N-H stretching absorptions in the regions 3 340 - 3 140 cm^-1 ^and 3 100 - 3 060 cm^-1 ^due to the bonded NH [20,24]. As the O-H stretching bands of the hydrogen-bonded hydroxyl display very strong absorptions in the region 3 400 - 3 200 cm^-1 ^[20,21,24], the N-H stretching absorptions of polypeptides therefore have been screened by the O-H stretching bands in this case. Thus, the absorption at 3 385 cm^-1 ^of the soluble D01 hydrolysate (Figure 2, curve 1) is assigned to the O-H stretching band (υ_O-H_) of the intermolecular, hydrogen-bonded hydroxyl of polysaccharides [24]. After the contact with Au(III), the filtrate showed the spectrum with a disappearance of the band at 3 385 cm^-1 ^(curve 2), owing to the hydroxyl-oxygen challenged with Au(III). The action of Au(III) on the oxygen incurs a red-shift of the O-H absorption, thereby leading to the loss of this band. Two other changes in the intensities of asymmetric and symmetric vibrations of the $\text{-C(}\underline{---}{\text{O)}}_{2}^{-}$ stretching frequencies of the carboxylate anion from the side chains at 1 587 and 1 406 cm^-1 ^[20,21,24] have occurred because of the binding of Au(III) to the oxygen of this group [22] (Figure 2). This is just similar to the case of the D01 biomass challenged with Au(III) mentioned above (Figure 1), only that the filtrate has succeeded in avoiding the obscuring of the carboxylate anion region by the absorptions of the insoluble substances to show the asymmetric $\text{-C(}\underline{---}{\text{O)}}_{2}^{-}$ absorption band at 1 587 cm^-1 ^more than the biomass. This typical complexation of the carboxylate anion with Au(III) results in a blue-shift of the asymmetric $\text{-C(}\underline{---}{\text{O)}}_{2}^{-}$ band at 1 587 cm^-1 ^and a red-shift of its symmetric absorption at 1 406 cm^-1 ^[22] respectively, thus causing a marked reduction in the intensity of the absorption at 1 587 cm^-1 ^and a clear shift of part of the absorption from 1 406 to 1 386 cm^-1^. This asymmetric absorption frequency of 1 587 cm^-1 ^is the stretching band of the $\text{-C(}\underline{---}{\text{O)}}_{2}^{-}$ resonating structure, being closely related to the property of O-Au(III) bond while the binding occurred. The resonance of the carboxylate anion causes a reduction in its bond order to result in a red-shift of the asymmetric frequency [22]; but a covalent bond should restrain the resonance, therefore the coordinate-covalent bond formation of O-Au(III) by the complexation of the $\text{-C(}\underline{---}{\text{O)}}_{2}^{-}$ anion with Au(III) incurs a blue-shift of this band. Similar changes for the same reason have also occurred in the circumstances of the soluble hydrolysates of R08, A09 and waste *S. cerevisiae *biomasses reacted with Au(III) respectively (Figures 3, 4 and 5). There are found each of the asymmetric $\text{-C(}\underline{---}{\text{O)}}_{2}^{-}$ stretching bands with obvious decreases in their intensities of the absorptions at 1 589 cm^-1 ^(Figures 3 and 4) and 1 587 cm^-1 ^(Figure 5), and each symmetric band with a clear red-shift of part of the absorption from 1 401 to 1 385 cm^-1 ^in Figure 3, 1 405 to 1 383 cm^-1 ^in Figure 4 and 1 410 to 1 392 cm^-1 ^in Figure 5, respectively. The IR frequency shifts of hydroxyl and carboxylate anion as well as both amide I and II bands (i.e. C=O and C-N-H groups of the peptide bond) could be correlated with the coordinate-covalent bond formation between Au(III) and donor atoms (O and/or N) of these groups [22,24].

Ordinarily, the carboxylate anion should be easily combined with the proton to form the carboxyl (-COOH) under acid condition; and the carbonyl absorption of the carboxyl, a most sensitive to IR absorption, must appear at 1 725 cm^-1^, but it wasn't found to occur after the filtrate of the D01 biomass in contact with Au(III) on the existence of proton (Figure 2, curve 2). And the absences of the carboxyl absorption also occurred in the soluble hydrolysates of R08, A09 and waste *S. cerevisiae *biomasses challenged with Au(III) at pH 3.0 respectively (Figures 3, 4 and 5). The results implies that the carboxylate anion of the filtrates reacted with the proton seems to be only through electrostatic attraction, i.e. a non-covalent interaction, and should still keep the ionic character in their respective biosorption systems. Naturally, there isn't the carbonyl absorption of the carboxyl of IR in each case. The findings just indicate that the proton appears not to have influenced the IR absorption of carboxylate anion or carboxyl under the circumstances. This evidence provides an important basis for discussion as to the mechanism of the redox reaction of Au(III) with the D01 biomass.

#### Bioreduction of Au(III) to Au(0)

As noted in the earlier studies [11,25], the market increase in the intensity of the hydroxyl of saccharides at 1 058 cm^-1 ^(δ_O-H _+υ_C-O_) in Figure 1, curve 2 is largely due to an increase in the free hydroxyl, thereby strongly suggesting that part of the polysaccharides on the cell wall of the biomass have been hydrolyzed to shorter saccharides of oligoses, dioses, monoses, etc. And most of the short saccharides should retain the monose group of the hemiacetalic hydroxyl, so that they still have the reducing ability like glucose and are referred to generally as reducing sugars. This result has further supported the existence of certain amount of the reducing sugars in this system. Since the proton of the system can't directly affect the IR absorption of the carboxyl in this case, one of the primary causes of the two distinctly enhanced intensities of the carboxyl absorptions at 1 725 cm^-1 ^(υ_C=0_) and 981 cm^-1 ^(δ_O-H_) in Figure 1 is thought to involve the oxidation of the reducing sugars to their corresponding acids by the noble-metal cation [11,25,26]. This inference has been confirmed by both XPS and IR characterizations of the interaction of glucose with Au(III) [11]. The mechanism of Au(III) interaction with the reducing sugars involves the rapid reduction of Au(III) to Au(I), followed by a slow reduction to Au(0). This redox reaction is expressed as follows:

$$\begin{array}{c} \text{Au}\left(\text{III}\right)+\text{R-CHO}+{\text{H}}_{\text{2}}\text{O}\to \text{Au}\left(\text{I}\right)+\text{R-COOH}+2{\text{H}}^{\text{+}}\\ \text{2Au}\left(\text{I}\right)+\text{R-CHO}+{\text{H}}_{\text{2}}\text{O}\to 2\text{Au}\left(0\right)+\text{R-COOH}+2{\text{H}}^{\text{+}} \end{array}$$

The biomass must have served as a catalyst as well as an electron donor in this redox reaction of normal temperature. In the course of Au(III) bioreduction, the free aldehyde group of the cyclic hemiacetalic hydroxyl from the various reducing sugars has been oxidized to the carboxyl with the bound Au(III) being in situ reduced to Au(0), thus leading to the marked increases in the intensities of the carboxyl absorptions at 1 725 and 981 cm^-1 ^respectively (Figure 1, curve 2). Whereas the carboxyl absorption is absent from the soluble hydrolyzed biomasses of D01, R08, A09 and waste *S. cerevisiae *reacted with Au(III) respectively (Figures 2, 3, 4 and 5, curves 2); besides the above cause that the proton of the systems seems not to have produced a direct effect on the carboxyl absorption in these cases, there should also be another reason that the redox reactions appear not to occur yet under the circumstances. Maybe these filtrates don't contain any enzymes that can enzymatically catalyze the bioreduction of Au(III).

#### Conformation-rearrangement of polypeptides by Au(III)-intervention

Interestingly, the soluble D01 hydrolysate challenged with Au(III) exhibits the IR spectrum with the amide I band splitting into two peaks of 1 658 and 1 634 cm^-1 ^(Figure 2, curve 2). This is ascribable to the interaction of the carbonyl-oxygen with Au(III) [23,24]. The carbonyl adsorption of the peptide bond in this case is not a pure C=O stretching mode but is appreciably coupled with the C-N stretch and to some extent with the NH bend [20,27]; hence the amide I band is believed to reflect the molecule-conformational information about the protein secondary structure involving α-helix, β-pleated sheet, β-turn, random coil and so forth [27]. In view of this reason, the absorptions of 1 658 and 1 634 cm^-1 ^are, respectively, attributed to the conformations of α-helix and parallel β-pleated sheet [23,24,27], being each characterized largely by the regular arrays of intra- and inter-molecular (intra- and inter-chain) hydrogen-bondings in polypeptides [24,28]. The frequency shift of a fair proportion of the absorption from 1 634 to 1 658 cm^-1 ^directly reflects that the action of Au(III) on the carbonyl-oxygen of the peptide bond appears to incur a fracture of much of the inter-chain hydrogen-bond linking adjacent polypeptide backbones and to bring the intra-chain hydrogen-bond formation, a corresponding conformation-transition (without a rupture of covalent bonds) from the parallel β-pleated sheet to the α-helices in polypeptide backbones thus happening. While the spectra of the D01 biomass and that reacted with Au(III) display the absorptions of the amide I band at 1 649 and 1 655 cm^-1 ^without a split respectively (Figure 1, curves 1 and 2), they each appreciably contain the different percentages of both the absorptions of 1 634 cm^-1 ^(due to parallel β-pleated sheet) and of 1 658 cm^-1 ^(due to α-helix). From the mathematical viewpoint, it was estimated about 37.5% parallel β-pleated sheet and 62.5% α-helix at 1 649 cm^-1^, and about 12.5% parallel β-pleated sheet and 87.5% α-helix at 1 655 cm^-1^. The result indicates that two thirds of the parallel β-pleated sheet has changed to the α-helices in polypeptides after in contact with Au(III) for 24 h. And a great deal of gold particles were formed then [8,17]. What has been mirrored about the reconformation of polypeptides by the D01 biomass is just the same as that by its soluble hydrolysate (Figure 2), only that the latter reflects the change in molecular conformation more sensitively than the former; this revealment by the filtrate complements that by the D01 biomass perfectly. Two other cases bearing strong resemblances to this are also watched in the filtrates of R08 and A09 biomasses and those challenged with Au(III) respectively (Figures 3 and 4). They each show the clear blue-shifts of a fair proportion of the absorptions from 1636 cm^-1 ^(due to the parallel β-pleated sheet) to 1 660 cm^-1 ^(due to the α-helix), thereby directly representing that lots of the β-pleated sheet has changed to the α-helices in polypeptides since the filtrates were in contact with Au(III) in each system. Another somewhat similar case is observed in the soluble hydrolysate of the waste biomass of *S. cerevisiae *and that reacted with Au(III) in Figure 5. It shows the absorptions of the υ_C=O _of the peptide bond at 1 631 and 1 609 cm^-1 ^(curve 1) arising, respectively, from parallel and antiparallel β-pleated sheets [27,28], characterized mostly by their different arrangements in polarity of peptide chains [28]. After in contact with Au(III), the filtrate displayed a spectrum with the clear shifts from 1 631 and 1 609 cm^-1 ^to 1 638 cm^-1 ^and one new shoulder peak occurred near 1 682 cm^-1 ^(curve 2); the lack of the absorption at 1 609 cm^-1 ^and the appearance of one band at 1 638 cm^-1 ^suggest a conformational transition from antiparallel to parallel β-pleated sheet in polypeptides. The band of 1 638 cm^-1 ^seems to contain about five sixths of parallel β-pleated sheet and one sixth of α-helix, estimated by the mathematical method; and the shoulder peak at 1 682 cm^-1 ^is assigned to the conformation of β-turn [23,27], also a helical circle, characterized largely by the periodic arrays of intra-chain hydrogen-bonding [28]. The findings reflect that part of the β-pleated sheet has been transformed into α-helices and β-turns of the circular forms since the filtrate of the waste biomass of *S. cerevisiae *was in contact with Au(III). Collino et al. [29] noted that the neutralized side-chains at low pH haven't led to an obviously conformational change in the participants of specific proteins. In view of this reason, the observed reconformations that occurred simultaneously with the changes in the arrays from inter- to intra-chain hydrogen-bonding don't arise from side-chain charge repulsion between adjacent cationic residues but from the intervention of Au(III). Collectively, the proteins of these biomasses on adsorbing Au(III) seem to show a strong tendency towards the transition of the molecular conformation from sheet of polypeptide backbone chains to separate-helical backbones in protein secondary structure; and this changing is bound to be accompanied by the alteration in side-chain steric positions of protein primary structure. The above evidence suggests that the conformation-lability is a common feature of the proteins and that the feature is associated with the Au(III)-binding effects. It is assumed that the conformation-labile (i.e. molecule-dynamic) property of the polypeptides favorably modulates the binding affinities by altering the side-chain positions with respect to the targets, which might lead to higher-affinity interactions of the helical polypeptides with Au(III), Au(I) and/or Au(0) surfaces [30,31].

#### Effects of molecular reconformation on Au(III) binding, nucleation and growth

α-helix, the commonest protein secondary structure, shows each of the helical circles to contain 3.6 amino-acid residues with a pitch of 0.54 nm between adjacent helical circles joined by intra-chain hydrogen-bonding that occurs between the hydrogen of the peptide bond and the oxygen of that of the fourth residue at N-terminal of the polypeptide backbone [28], i.e. -N-H...O=C- (hydrogen-bonding expressed in dotted line), and has the regular, helical conformation-pattern; it seems only one fourth of -C=O and H-N- groups of the peptide bond have shares in the hydrogen-bonding and three fourths of those are on free state. For β-turn it bears regular circles of four amino-acid residues each and also displays helical backbone with the periodic arrays of intra-chain hydrogen-bonding [28]; similarly, it appears three fourths of -C=O and H-N- groups of the peptide bond are unbound. And for β-pleated sheet, the second most content in protein secondary structure, it includes parallel and anti-parallel patterns with periodic arrays of inter-chain hydrogen-bonding that occurs between -N-H and O=C- groups of the peptide bond from each of the adjacent backbones, and has the conformation of regular pleat-sheet [28]; it appears almost all -C=O and H-N- groups of the peptide bond participate in the hydrogen-bonding, so that there seems the lack of the free -C=O and H-N- groups in β-pleated sheet. Both α-helix and β-turn of circular patterns have shown their higher frequencies of the carbonyl (-C=O) absorption of the peptide bond in IR than the β-pleated sheet just because each of the former cases possesses more unbound carbonyl than the latter. The reconformation of polypeptides from pleated sheet to helical circles is therefore thought to free much -C=O and H-N- groups of the peptide bond from the hydrogen-bonding as well as to alter side-chain steric positions for favoring the accessibility of peptide bond and amino-acid residues to the targets. The investigation of these filtrates reacted with Au(III) (Figures 2, 3, 4 and 5) by IR indicates that much Au(III) has been bound to peptide bond and amino-acid residues and that the Au(III) bound by oxygen and/or nitrogen of the peptide bond has been carried from β-pleated sheet into α-helices and β-turns of circular conformations. It seems that the polypeptides make the opportunities for Au(III) getting higher-affinity bindings from the unbound -C=O and H-N- groups of the peptide bond and from the $\text{-C(}\underline{---}{\text{O)}}_{2}^{-}$ group of side-chains via the molecular reconformations, thereby impelling Au(III) to speciate with these functional groups. And certain binding speciations of gold with oxygenous- and nitrogenous-active-groups in cell wall biopolymers of the biomass have been deduced from the IR experimental evidence. The active binding sites of Au(III) to the biomass, such as hydroxyl of polysaccharides, carboxylate ion of amino-acid residues, peptide bond, etc. could much probably serve as the nucleation positions for Au(0) nuclei growth. For the resulting binding species of Au(0), the coordinating atoms (O and/or N) might greatly contribute to a better regulation of Au(0) nucleation and crystal growth; and the surrounding unbound oxygen and/or nitrogen could still much probably adsorb alone or together onto different parts of the Au(0) nucleus and/or nanoparticle surface, which might lead to the competitive adsorption-nucleation in polypeptide-mediated biomineralization [32] and/or to the regulation of surface-reaction-limited growth of nanocrystal [33], the particle size and shape being thus governed. In view of these properties peculiar to the biomacromolecules, combinatorial approaches [34-36] have been used to identify peptides that selectively recognize inorganic surfaces; in some cases these peptides serve as templates for inorganic deposition, offering a way for spatially controlling inorganic nucleation and crystal growth. A new peptides' adsorption-nucleation model put forward by Muthukumar [32] will undoubtedly make a significant contribution to the direct selections of crystal morphologies and growth kinetics in the course of polypeptide-mediated biomineralization.

From the viewpoint of the molecular conformation that the β-pleated sheet has backbones similar to the linear with the side-chains positioned closer together and that both the circular forms of α-helix and β-turn present their helical backbones with the side-chains separated from one another compared to the pleated sheet, it seems that the Au(0) nanoparticles bound on these side-chains of the helical patterns couldn't likely polymerize and should keep stability more easily than those of the β-pleated sheet, besides that the helical circles might create more accessible side-chain regions for challenged with Au(III) effectively. In addition, the helical forms possessing much unbound -C=O and H-N- groups of the peptide bond might be reasonably expected to be more favorable than β-pleated sheet (without the free -C=O and H-N- groups of the peptide bond) for Au(0) particles obtaining better regulation and shelter besides for Au(III) getting higher-affinity action from polypeptides. Hence this molecular conformation-rearrangement in polypeptide backbones from the pleated sheet to the helical versions appears to construct more advantageous conditions to suit the needs of the Au(III) intervention and to facilitate the regulation of Au(0) nanoparticles. Also, the conformation-labile polypeptides may enhance their adaptability to interfacial features that exist on gold surfaces (e.g. surface topology, interfacially bound water layers); and this conformation-instability of proteins involved in mineral formation/modification may be regarded as a driving force for polypeptide-mediated crystal growth and regulation [31,37,38]. It might therefore be expected that most of the Au(0) on cell wall surfaces should well nucleate and that the growth of Au nuclei should be modulated and regulated by polypeptides, homogeneous gold nanoparticles would thus be obtained. In reality, quite a few uneven gold nanoparticle distributions have been observed in the earlier investigations of the interactions of Au(III) with D01 [17] and waste *S. cerevisiae *[8] biomasses. There seem two possibilities that account for the occurrence of the larger sizes of the particles in the systems. One appears due to a decrease in the pH of the biosorption system, because both the processes of adsorption and reduction of Au(III) by the biomass usually result in the releases of the proton [6,25,26] and further the acidification of the present system. A drop in the pH value from 3 to 1.96 occurred in a matter of one hour after the biosorption, indicating that the concentration of the proton has reached more than ten times as thick as that at the beginning; and it may be going on falling with the proceeding of the biosorption. Thus, the excessive proton could occupy the binding sites of Au(III), Au(I) and Au(0), especially the Au(0) position, causing part of the Au(0) to be separated from the biomass and to be no longer sheltered and regulated by the biomacromolecules. In this case, the fallen free-nanoparticles may likely gather one another under the circumstances of Brownian motion. And moreover, the excessive proton could also seize the free oxygen and/or nitrogen atoms of the active groups of the proteins, leading to hamper the polypeptides' competitive adsorption-nucleation models [32] that will control both size and shape of Au(0) nanoparticles. To avoid the adverse effect of the pH on Au adsorption, nucleation and crystal growth, the adjustment of the pH may have to be timely made and the value had better be maintained at pH 3.0 during the biosorption process. The other likelihood for the polymerization of gold nanoparticles seems the occurrence of the Brownian motion of the biomass in the systems. This factor can't be ignored although the motion is invisible to the naked eyes. The cells with sizes of 0.5 - 1 (diameter) × 1 - 3 (length) (μm) have been unceasingly shocked by far smaller liquid medium molecules making an uninterrupted thermal motion. Thus, part of the crystals without getting better regulation and shelter on cell surfaces may form twinned crystals or develop multiple twins, or fall off the cell walls, by the impact of the Brownian motion of the biomass. To reduce the unfavorable factors to the minima, the initial concentration of Au(III) must be prepared in a suitable range; the reaction time should be kept within the time frame of the experiment and so forth; and an appropriate capping ligand, such as dodecanethiol (C_12_H_25_SH) [39], may be considered being used for sheltering and stabilizing the Au(0) nanoparticles still further. Obviously, much work needs getting on to find out the most suitable conditions for the biosynthesis of homogeneous gold nanoparticles and to develop new methodologies to facilitate the biopreparation process and its optimization.

## Conclusions

The interaction of Au(III) with *Bacillus megatherium *D01 biomass on a molecular level has been further characterized by IR spectrometry in this paper. The findings indicate that the adsorption of Au(III) onto the biomass is mainly through an ion-exchange mechanism; and that this sorbing is closely followed by the coordinations of Au(III) with the active groups of the biomass, namely the coordinate-covalent bond formation between Au(III) and the donor atoms (O and/or N) of these groups, such as hydroxyl, carboxylate ion, peptide bond, etc. The evidence of the bioreduction of Au(III) to elemental Au(0) at normal temperature and the formation of Au(0) nanoparticles within a short time shows that the biomass itself must have fulfilled the role as a catalyst as well as playing an important part in regulating Au nanoparticles besides as an electron donor during this biosorption. A further investigation into the interactions of Au(III) with each of the filtrates of D01, R08, A09 and waste *S. cerevisiae *biomasses strongly suggests an essential biomacromolecule-characteristic that appears the binding of Au(III) to the oxygen of the peptide bond has led to a significant transition, in molecular conformations of protein secondary structure, from sheet of polypeptide backbones to separate-helical chains, namely a reconformation from β-pleated sheet to α-helices and/or β-turns; and this changing is accompanied by the alteration, of protein primary structure, in side-chains' accessibility to Au(III), as well as by the occurrence, in the peptide bond, of much unbound -C=O and H-N- groups that have been freed from the inter-molecular hydrogen-bonding of the β-pleated sheet and carried on the helical versions for facilitating binding Au(III). The reconformations of protein primary and secondary structures by the intervention of Au(III) seem to construct more accessible side-chain and peptide bond regions for reacting upon Au(III) effectively and to create more favorable conditions for polypeptides and their side-chains regulating and stabilizing Au(0) nanoparticles. It appears that the polypeptides are activated by Au(III) via the molecular reconformation and in turn act on Au(III) positively and produce important effects on Au(0) nucleation and crystal growth.

The revealment of the interactions of Au(III) with the microbial biomasses could provide useful information for developing new methodologies to facilitate both the biosynthesis of new advanced gold nanostructured materials and the improvement of the biopreparation of high-dispersively supported gold catalyst.

## Authors' contributions

YY carried out the XRD examination, and participated in both analysis and interpretation of XRD patterns. QL and ZX carried out the biosorption examinations, and participated in the analyses of adsorptive efficiency and capacity of the D01 biomass for Au(III). MW carried out the UV-vis examination and participated in the analysis of glucose content of the D01 biomass. ZL conceived of the study, carried out the IR examination, performed the synthetic analysis and drafted the manuscript. All the authors have read and approved the final manuscript.

## Note

*The figure was once published in the manuscript entitled "Spectroscopic characterization on interaction of gold (Au^3+^) biosorption by Bacillus megatherium D01" with the journal of *Acta Chim Sin *in 2004, **62 **(18): 1829-1834. (Ref. 10)
